# Association of Peer Network with Childhood Obesity in DECIDE-Children Program

**DOI:** 10.3390/nu15194154

**Published:** 2023-09-26

**Authors:** Ping Li, Jinlang Lyu, Shuang Zhou, Zheng Liu, Xiangxian Feng, Yi Lin, Aiyu Gao, Fang Zhang, Haijun Wang

**Affiliations:** 1 Department of Maternal and Child Health, School of Public Health, Peking University, Beijing 100191, China; 2 Peking University Health Science Center-Weifang Joint Research Center for Maternal and Child Health, Weifang 261000, China; 3 Department of Preventive Medicine, Changzhi Medical College, Changzhi 046000, China; 4 Urumuqi Primary and Secondary School Health Care Center, Urumuqi 830003, China; 5 Dongcheng Primary and Secondary School Health Care Center, Beijing 100010, China; 6 Mentougou Primary and Secondary School Health Care Center, Beijing 102300, China

**Keywords:** social networking, pediatric obesity, popularity, centrality

## Abstract

Some studies have found associations between the peer network and childhood obesity. The present study aimed to analyze the association of the peer network with obesity-related cognition, behaviors and adiposity indicators, and explore whether peer network influences the effect of a childhood-obesity intervention. Based on DECIDE-Children, 1392 children’s friendship nominations within the class were collected and peer network indicators including the network size, network density, and in- and out-degree centrality were calculated. The linear mixed model was used to analyze the association between peer network indicators and children’s cognition, behaviors and adiposity indicators (body mass index (BMI), BMI z score, the prevalence of overweight and obesity). Children with a higher in-degree centrality had 34.4% (95%CI: 17.4% to 48.1%) lower risk of overweight or obesity. The baseline degree centrality was inversely associated with the BMI and BMI z score at the end of the trial. For each unit increase in in-degree centrality at baseline, the BMI at the end of the trial decreased by 0.047 (95%CI: 0.015 to 0.080), and the BMI z score decreased by 0.015 (95%CI: 0.003 to 0.028). Children’s popularity reflected by centrality in their peer network was associated with cognition, behaviors, and adiposity indicators. Future childhood-obesity intervention research could pay more attention to socially inactive children.

## 1. Introduction

Individuals are embedded in thick networks of social relationships and interactions. Social networks represent relatively stable systems composed of relationships among individuals (e.g., friendship), and reflect an individual’s social environment [1]. In social-network analysis, individuals in social networks are “nodes”, and relationships among individuals are the “edges” among the nodes. These social relationships can be quantified in different ways, focusing on either the individual’s position within the network or the network as a whole. One way to quantify the individual’s position is by using the indicator of centrality. Centrality is one of the most commonly used network indicators in childhood health research [2]. Centrality indicates the prominent individuals who are extensively involved in relationships with other network members and represents the extent to which a person inhabits a prestigious or critical position in the network. Degree centrality is measured as the number of existing edges (relationships) connected to one node (individual), that is the number of links to and from one person [1]. The centrality is a purely structural measure of popularity in a network, that is, the more connections or centrality reflects more popularity.

Traditional epidemiological research has mainly focused on health-risk behaviors of individuals in a group, while the network theory suggests that individuals’ health and health-related behaviors are affected by the embedded social network [3]. A peer network is a type of social network among children based on collective learning and life. Harris’s group socialization theory states that peer groups are the main environmental motivation for children’s socialization development, and children adjust their behaviors according to the behavioral norms of peers [4]. Children’s health and health behaviors are influenced not only by their own behavior and their social physical environment, but also by their social environment, and the peer network is one of the important social networks in children.

To date, some observational evidence has supported the idea that the social environment is one of major determinants of health and health-related behaviors, and highlighted the role of the peer network in the children’s health. Children’s activeness or popularity in a peer network might affect their health-related behaviors, such as sleep, physical activity and sedentary behaviors [5,6]. Some studies have found that active social competence might contribute to a healthy nutritional status and weight-loss behaviors [7,8,9,10]. Furthermore, a high popularity, reflected by centrality during childhood, significantly predicted reduced cardiometabolic risk during adulthood [11]. In conclusion, the previous study indicated that the activeness or popularity of children in a peer network was positively associated with the children’s healthy obesity-related behaviors and healthy weight statuses. Despite prior observational studies highlighting the role of the peer network in childhood obesity [2,12], to the best of our knowledge, no study has explored whether and how the peer network influences the effect of a childhood-obesity intervention.

Based on a multifaceted intervention program for preventing obesity in primary school children in China, the present study aimed to analyze the association of the peer network with obesity-related cognition, behaviors and adiposity indicators, and explore whether centrality in a peer network influences the effect of a childhood-obesity intervention (i.e., adiposity indicators at the end of the trial), in order to provide scientific evidence for the future childhood-obesity intervention strategies.

## 2. Methods

### 2.1. Study Design and Participants

The participants in this study were from a cluster randomized controlled trial named Diet, Exercise and Cardiovascular Health-Children (DECIDE-Children) conducted in 3 socioeconomically distinct regions from the eastern (Beijing), central (Changzhi, in Shanxi Province), and western (Urumqi, in Xinjiang Province) parts of China from September 2018 to June 2019. There were 705 grade 4 students (about 10 years old in the Chinese school system) from 12 intervention schools and 687 from 12 control schools. Children of this age have the ability to complete questionnaires. Based on the socio-ecological model, dietary and exercise interventions for children were carried out in the intervention group at the school, family and individual levels. For the school level, we developed school obesity prevention policies including physical education sessions, healthy food environment, and health education sessions for teachers and children. For the individual level, we promoted children’s physical exercise at school and at home and recommended for them not to eat fried food, snacks, fast food, etc. For the family level, we conducted health education sessions for parents. In addition, an application on the mobile phone of the parents (named Eat Wisely and Move Happily) was used to conduct the regular monitoring and feedback of children’s diet and exercise behaviors and adiposity indicators, and promote the family involvement in the childhood-obesity intervention. Details of sampling and interventions can be found in previously published articles [13,14]. Ethical approval was granted by the Peking University Institutional Review Board (IRB00001052-18021), and the informed consent of the children and their parents was obtained.

### 2.2. Outcome Assessment

The children’s obesity-related cognition and behaviors were collected through questionnaires at the baseline and at the end of the trial, which included (1) the children’s correct rate of answering obesity-related questions; (2) dietary habits (without the intake of sugar-sweetened beverages, snacks, fried food, western fast food, and including eating breakfast every day); (3) exercise behavior (the number of days with ≥1 h moderate-to-vigorous physical activity per week); and (4) screen behavior (average daily screen time ≤ 2 h). In order to comprehensively evaluate obesity-related cognition and behaviors, the scores of the above 4 dimensions were added to obtain a score of Weight-control-related Cognition and Behavior (WCB), with each dimension scored according to a full score of 5. A higher WCB score indicated healthier obesity-related cognition and behaviors in a child [15].

The adiposity indicators in this study included the body mass index (BMI) and BMI z score. Children’s anthropometric measures at the baseline and at the end of the trial were collected by trained personnel, using identical devices and standardized forms referring to standard methods and procedures. The BMI z score was calculated according to World Health Organization (WHO) standards [16]. Overweight and obesity were evaluated using age- and sex-specific BMI percentiles according to the Chinese reference [17].

### 2.3. Assessment of Peer Network

Children were asked to fill out questionnaires at school at baseline for collecting data on children’s peer networks. Each child was asked to nominate his/her friends within the same class who met at least two of the following five conditions: they (1) play together almost every day during recess, (2) often play or do homework together after school (>3 days/week), (3) often share snacks and toys, (4) often share extracurricular books, or (5) often go to school or go home after school together.

The children’s friendship-nomination data were organized into a binary relational matrix, with the number of rows and columns equal to the total number of respondents in the class, and Ucinet 6 software was used to calculate peer network indicators at the individual and group levels. Peer network indicators included the network size, network density (group level) and degree centrality (individual level). Specifically, the network size was the number of children in each class, indicating the size of the relationship network within the class. The network density was the existing total number of friendship connections (ties) divided by the total possible number of ties within the same class, indicating the degree to which individuals in a peer network are connected to each other. Degree centrality was the total number of friends one child had. This could act as a measure of popularity or socially activeness in a peer network. Based on the direction of friendship nomination, the degree centrality was classified as out-degree and in-degree centrality in the social network analysis. Specifically, the out-degree centrality was the number of friendship nominations one child sent to others. And the in-degree centrality was the number of relationship nominations one child received from other classmates in their peer network.

### 2.4. Statistical Analysis

Categorical variables were presented as numbers and percentages (%), and continuous variables with normal distribution were presented as means and standard deviations (SD). Basic characteristics were compared using student’s *t* test for continuous data, with a Chi-square test for categorical variables. The association of the peer network with WCB and adiposity indicators at baseline was assessed using linear mixed models that included class-level random intercepts to account for the clustering effect of children nested within the same class, with adjustment for age, gender, region, etc. General linear models and linear mixed models were used to explore the association of baseline degree centrality with adiposity indicators at the end of the trial, with adjustment for corresponding adiposity indicators at baseline, group, age, gender, region, etc. For sensitivity analysis, we further adjusted for parenting styles (whether parents limited children’s screen time), psychological factors (self-efficacy in weight loss), family dynamics (family support for the intervention program), and school environments (school physical-education environment). The results were considered statistically significant at a two-sided *p* < 0.05. Statistical analysis was carried out using R software (version 4.2.2). Network properties were calculated in Ucinet software (version 6.0).

## 3. Results

### 3.1. General Characteristics of Children

A total of 1392 children were involved in this study. The control group and intervention group had similar sociodemographic characteristics at baseline. There were no significant differences in the adiposity indicators and degree centrality between the two groups, while the difference in network size and network density was statistically significant (Table 1).

### 3.2. Association of Peer Network with WCB and Adiposity Indicators

We found that out-degree centrality was positively associated with the WCB and negatively associated with the BMI z score at baseline. For each unit increase in out-degree centrality (i.e., the child nominated one more friend), the WCB increased by 0.172 (95%CI: 0.093 to 0.250), and the BMI z score decreased by 0.042 (95%CI: 0.001 to 0.083) in the children. The in-degree centrality was positively associated with the WCB and negatively associated with the BMI and BMI z score. For each unit increase in in-degree centrality (i.e., the child received one more friendship nomination), the WCB increased by 0.153 (95%CI: 0.064 to 0.241), BMI decreased by 0.184 (95%CI: 0.072 to 0.290), and BMI z score decreased by 0.066 (95%CI: 0.024 to 0.107). In addition, children with higher in-degree centrality had a lower risk of overweight or obesity (65.6%, 95%CI: 51.9% to 82.6%). And the network size was positively associated with BMI, but the association was no longer statistically significant after adjusting for parental educational level, parental nutritional status, etc. There were no statistically significant associations between the network density and WCB and adiposity indicators, nor between the network size and adiposity indicators (See Table 2). We performed an analysis of the four obesity-related cognition and behavior factors included in the WCB separately, including knowledge, diet, and screen and exercise behaviors. From the analysis results, high out-degree centrality was associated with healthy knowledge and exercise behavior. And high in-degree centrality was associated with healthy knowledge and screen behavior. In general, the degree centrality was positively associated with healthy obesity-related cognition and behaviors, which was consistent with our results of the association between peer network indicators and WCB. The sensitivity analysis additionally adjusting for other factors (whether parents limited children’s screen time, self-efficacy in weight loss, family support for the intervention program, school physical-education environment) showed similar results (See Table 3).

### 3.3. Association between Baseline Degree Centrality and Adiposity Indicators at the End of the Trial

The results of the linear mixed models showed that the baseline degree centrality was inversely associated with BMI and BMI z score at the end of the trial. Specifically, for each unit of increase in out-degree centrality at baseline (i.e., the child nominated one more friend), the BMI at the end of the trial decreased by 0.042 (95%CI: 0.014 to 0.071), and the BMI z score decreased by 0.018 (95%CI: 0.007 to 0.029) in children; for each unit of increase in in-degree centrality at baseline (i.e., the child received one more nomination), the BMI at the end of the trial decreased by 0.047 (95%CI: 0.015 to 0.080), and the BMI z score decreased by 0.015 (95%CI: 0.003 to 0.028) in children (See Table 4). The sensitivity analysis additionally adjusting for socioeconomic status (parental educational level), genetic predisposition (parental nutritional status), parenting styles (whether parents limited children’s screen time), psychological factors (self-efficacy in weight loss), family dynamics (family support for the intervention program), and school environment (school physical-education environment) showed similar results (See Table 5 and Table 6). In addition, the reduction in the BMI of the children with higher in-degree centrality (above median) was larger than those with lower in-degree centrality (−0.03 vs. 0.10; *p* = 0.030). The reduction in BMI of the children with higher out-degree centrality (above median) was also larger than those with lower out-degree centrality (−0.03 vs. 0.10; *p* = 0.020).

## 4. Discussion

This study found that the children’s degree centrality in their peer network was associated with obesity-related cognition, behaviors and adiposity indicators. Those socially active children in the class tended to have healthy obesity-related cognition, behaviors and adiposity indicators. We also found that in-degree centrality was more closely associated with childhood obesity than out-degree centrality. This may be because a child’s in-degree centrality (i.e., the number of friendship nominations a child received from others) could more accurately reflect the popularity of the child within the peer network compared with out-degree centrality. Furthermore, the degree centrality of the children was inversely associated with the adiposity indicators at the end of the trial. In addition, children with a higher degree centrality had a larger reduction in their BMI and BMI z score in the obesity prevention program, indicating that more popular or central children in the class benefited more from the prevention program.

The degree centrality in this study reflected the social status of children in the social environment within the class, indicating the degree to which children were welcomed by their peers (i.e., popularity or activeness). This study found out the association between the degree centrality and cognition, behaviors and adiposity indicators in the children, which was consistent with previous observational findings. It was found that there was a significant difference in centrality between the overweight and normal-weight children, with overweight children receiving fewer friendship nominations and less popularity among peers, suggesting the social marginalization of overweight children [18,19,20,21]. In addition, it was reported that children with active peer relations and social competence were less likely to be overweight or obese, while poor social competence could significantly predict a subsequent increased BMI and increased risk of obesity [7,8,9,10]. The explanation could be that children who were less socially integrated and unpopular with peers may avoid engaging in activities with classmates and probably exhibit overeating and inactivity, for example, watching television and finding solace for loneliness in calorie-rich foods, leading to a higher risk of weight gain [8].

This study identified that the degree centrality played a role on childhood obesity, and found that children with a higher degree-centrality had lower BMI and BMI z score at the end of the trial, indicating that children who were connected to their peers in class benefited more from the obesity intervention. This was consistent with the results of the School-EduSalt trial, which found out that children with more friends tended to have a larger salt intake reduction (β = 0.5, *p* = 0.044) [22], and suggested that popular children were likely to benefit more from lifestyle interventions. In addition, an observational study found that obese children with higher social competence were more likely to lose weight (OR = 1.43, *p* < 0.05) [8]. Another observational study found that children in less-centrally located social roles tended to have less physical activity than those with more-centrally located social positions [23]. Furthermore, it was found that adults who were active in social networks had a higher self-efficacy, less sedentary behaviors and more weight loss in adulthood obesity interventions [24,25]. However, it is worth noting that there were still some differences between the two adult studies and our study. The group-based adult obesity intervention [24] and the social media-based weight-loss intervention [25] were developed to intentionally create peer-to-peer interaction to spread new behaviors within groups of adults. However, our childhood-obesity intervention program focused on the individual, family and school environment. In addition, social relationships were assessed with advice seeking [24] or posts, comments and reactions [25] within adults, while we assessed peer relations with friendship nominations, which are usually used among children [26,27]. Children with higher centrality or popularity benefited more from the intervention programs, possibly because they were provided with more opportunities to gain peer support, had a better sense of belonging to the class, and were more likely to get involved in intervention programs and to be role models for other children. In addition, popular children were more integrated with peers, prone to have stronger prosocial behaviors, self-esteem and empathy, and more socially cooperative, and, thus, more motivated to participate in programs, and tended to adopt new behaviors more quickly [28,29,30,31]. Finally, this was also due to significant differences in neurocognitive activity between children with higher and lower centrality. For example, among popular children, the caudate nucleus is more active and they are neurologically more similar with general peers [32,33].

The strength of this study is that it is the first to explore the role of the peer network on childhood obesity, based on a well-designed randomized controlled trial that successfully decreased children’s BMI and BMI z score. We found that children with higher degrees of centrality had lower BMI or BMI z score at the end of the trial, which was consistent with findings in adult obesity intervention studies and other childhood behavioral interventions and observational studies. In addition, centrality was divided into in-degree and out-degree centrality based on peer network data, and it was revealed that in-degree centrality was more closely associated with children’s cognition, behaviors and adiposity indicators than out-degree centrality. For the association between the peer network and childhood obesity, the present prospective obesity intervention trial with a plausible temporal relationship overcame the possibility of reverse causality found in previous cross-sectional studies. And covariates such as the family support, socioeconomic status, self-efficacy, and class clustering effect were taken into account, making the results more reliable. However, the study had several limitations. First, the information about children’s obesity-related behaviors, including diet, exercise, and screen behaviors, were self-reported, and possibly affected by social desirability bias and recall bias. However, we found that the change in behavioral measures paralleled the changes in objective adiposity indicators, and the peer network indicators were inversely associated with adiposity indicators. Second, we identified friendships within the same classes, and lacked data of children’s other friends out of the class. Future studies are needed to identify a wider range of children’s friends to estimate children’s peer networks more comprehensively. Third, we explored the short-term association between the peer network and adiposity indicators. Future research could further explore the association between the peer network position in childhood and adulthood health outcomes. Lastly, although the research participants in the present study were from three socioeconomically distinct regions in China, from the eastern, central, and western parts, and included schools in rural and urban areas, the study’s applicability to larger populations and diverse cultural settings should also be considered with caution.

## 5. Conclusions

This study found that children with more friends in their peer networks tended to have healthy cognition and behaviors, and lower BMI. In addition, children who were more active and popular in their peer networks tended to benefit more from the intervention program. Future childhood-obesity intervention research should pay more attention to socially inactive children and explore how to enhance obesity intervention effects among these children.

## Figures and Tables

**Table 1:** General characteristics of the children.

Characteristics	Intervention (*n* = 705)	Control (*n* = 687)	*p*
Age (years)	9.62 ± 0.35	9.63 ± 0.37	0.482
Boy, *n* (%)	353 (50.07%)	364 (52.98%)	0.301
Anthropometric measures			
WCB	13.53 ± 3.07	13.64 ± 2.89	0.502
BMI	18.54 ± 3.70	18.76 ± 3.72	0.276
BMI z score	0.70 ± 1.43	0.79 ± 1.45	0.247
Overweight/obesity	266 (38.78%)	296 (43.79%)	0.068
Peer network indicators ^a^			
Network size	33.28 ± 8.47	32.26 ± 8.55	0.026 *
Network density	0.08 ± 0.02	0.09 ± 0.02	<0.001 *
Out-degree centrality	2.43 ± 1.98	2.37 ± 2.04	0.595
In-degree centrality	2.35 ± 1.81	2.24 ± 1.81	0.295

^a^ Measures at cluster (class) level. * *p* < 0.05. Abbreviations: WCB, Weight-control-related Cognition and Behavior; BMI, body mass index.

**Table 2:** Association of peer network with WCB and adiposity indicators at baseline.

Peer Network Indicators	WCB and Adiposity Indicators	Model 1 ^a^		Model 2 ^b^	
		β/OR (95%CI)	*p*	β/OR (95%CI)	*p*
Network size	WCB	−0.008 (−0.075, 0.059)	0.823	0.009 (−0.060, 0.078)	0.811
	BMI	0.069 (0.024, 0.114)	0.003 *	0.052 (−0.001, 0.103)	0.067
	BMI z score	0.017 (0.000, 0.035)	0.054	0.011 (−0.008, 0.031)	0.282
	Overweight/obesity ^c^	1.020 (0.751, 1.382)	0.899	1.078 (0.728, 1.592)	0.707
Network density	WCB	−3.343 (−22.215, 15.530)	0.737	−3.913 (−23.867, 16.076)	0.711
	BMI	−3.700 (−16.936, 9.530)	0.588	−12.466 (−28.762, 3.908)	0.154
	BMI z score	−0.882 (−5.997, 4.232)	0.736	−3.844 (−9.895, 2.351)	0.241
	Overweight/obesity ^c^	0.942 (0.724, 1.224)	0.653	0.908 (0.653, 1.260)	0.563
Out-degree centrality	WCB	0.172 (0.093, 0.250)	<0.001 *	0.121 (0.034, 0.206)	0.006 *
	BMI	−0.066 (−0.162, 0.031)	0.179	−0.096 (−0.202, 0.010)	0.076
	BMI z score	−0.033 (−0.070, 0.005)	0.086	−0.042 (−0.083, −0.001)	0.048 *
	Overweight/obesity ^c^	0.884 (0.706, 1.107)	0.282	0.835 (0.633, 1.101)	0.202
In-degree centrality	WCB	0.153 (0.064, 0.241)	<0.001 *	0.124 (0.027, 0.220)	0.012 *
	BMI	−0.184 (−0.290, −0.072)	<0.001 *	−0.212 (−0.329, −0.094)	<0.001 *
	BMI z score	−0.066 (−0.107, −0.024)	0.002 *	−0.077 (−0.123, −0.032)	0.001 *
	Overweight/obesity ^c^	0.656 (0.519, 0.826)	<0.001 *	0.679 (0.510, 0.901)	0.007 *

^a^ Model 1 adjusted for age, sex, region, and class clustering effects. ^b^ Model 2 further adjusted for parental educational level, parental nutritional status (whether overweight/obese), and birth weight. ^c^ The peer network indicators were classified according to the median, with the lower group as the reference group; no adjustment for class clustering effect owing to it being a singular model. * *p* < 0.05. Abbreviations: WCB, Weight-control-related Cognition and Behavior; BMI, body mass index.

**Table 3:** Sensitivity analysis of the association between the peer network and WCB and adiposity indicators at baseline.

Peer Network Indicators	WCB and Adiposity Indicators	Model 1 ^a^		Model 2 ^b^		Model 3 ^c^		Model 4 ^d^	
		β/OR (95%CI)	*p*	β/OR (95%CI)	*p*	β/OR (95%CI)	*p*	β/OR (95%CI)	*p*
Network size	WCB	−0.015 (−0.081, 0.051)	0.678	−0.015 (−0.081, 0.051)	0.666	−0.009 (−0.076, 0.059)	0.806	−0.005 (−0.072, 0.062)	0.887
	BMI	0.077 (0.031, 0.124)	0.001 *	0.077 (0.032, 0.123)	0.001 *	0.072 (0.026, 0.117)	0.002 *	0.072 (0.027, 0.118)	0.002 *
	BMI z score	0.021 (0.003, 0.039)	0.020 *	0.020 (0.003, 0.038)	0.024 *	0.019 (0.001, 0.036)	0.041 *	0.018 (0.001, 0.036)	0.043 *
	Overweight/obesity ^e^	1.035 (0.755, 1.417)	0.830	1.080 (0.793, 1.469)	0.623	1.025 (0.753, 1.393)	0.873	1.062 (0.780, 1.443)	0.702
Network density	WCB	−1.193 (−19.993, 17.630)	0.906	−4.668 (−23.330, 14.009)	0.641	−3.952 (−23.021 15.131)	0.699	−4.413 (−23.383, 14.574)	0.668
	BMI	−4.778 (−18.548, 8.991)	0.503	−3.980 (−17.382, 9.433)	0.571	−4.434 (−17.847, 8.982)	0.522	−4.167 (−17.565, 9.231)	0.543
	BMI z score	−1.544 (−6.886, 3.797)	0.572	−1.029 (−6.210, 4.152)	0.698	−1.264 (−6.449, 3.921)	0.634	−1.080 (−6.258, 4.0980)	0.683
	Overweight/obesity ^e^	0.920 (0.701, 1.207)	0.548	0.921 (0.705, 1.202)	0.543	0.920 (0.705, 1.199)	0.536	0.920 (0.706, 1.199)	0.538
Out-degree centrality	WCB	0.160 (0.080, 0.239)	<0.001 *	0.139 (0.061, 0.217)	<0.001 *	0.167 (0.088, 0.245)	<0.001 *	0.173 (0.094, 0.251)	<0.001 *
	BMI	−0.077 (−0.174, 0.022)	0.124	−0.069 (−0.165, 0.030)	0.166	−0.069 (−0.166, 0.028)	0.162	−0.064 (−0.161, 0.032)	0.193
	BMI z score	−0.035 (−0.073, 0.003)	0.068	−0.034 (−0.071, 0.004)	0.081	−0.033 (−0.071, 0.004)	0.084	−0.032 (−0.069, 0.005)	0.092
	Overweight/obesity ^e^	0.874 (0.693, 1.101)	0.253	0.892 (0.710, 1.121)	0.328	0.876 (0.699, 1.098)	0.250	0.886 (0.707, 1.109)	0.291
In-degree centrality	WCB	0.136 (0.046, 0.225)	0.003 *	0.138 (0.050, 0.224)	0.002 *	0.152 (0.063, 0.240)	0.001 *	0.153 (0.064, 0.241)	0.001 *
	BMI	−0.184 (−0.290, −0.068)	0.001 *	−0.185 (−0.290, −0.071)	0.001 *	−0.185 (−0.289, −0.073)	0.001 *	−0.178 (−0.282, −0.068)	0.001 *
	BMI z score	−0.062 (−0.104, −0.020)	0.004 *	−0.065 (−0.106, −0.023)	0.002 *	−0.066 (−0.107, −0.024)	0.002 *	−0.064 (−0.105, −0.023)	0.002 *
	Overweight/obesity ^e^	0.648 (0.510, 0.822)	<0.001 *	0.662 (0.523, 0.837)	<0.001 *	0.652 (0.516, 0.823)	<0.001 *	0.662 (0.524, 0.835)	<0.001 *

^a^ Model 1 adjusted for age, sex, region, and class clustering effects, and whether parents limited children’s screen time. ^b^ Model 2 adjusted for age, sex, region, and class clustering effects, and self-efficacy in weight loss. ^c^ Model 3 adjusted for age, sex, region, and class clustering effects, and family support for the intervention program. ^d^ Model 4 adjusted for age, sex, region, and class clustering effects, and school physical education environment. ^e^ The peer network indicators were classified according to the median, with the lower group as the reference group; no adjustment for class clustering effect made owing to singular model. * *p* < 0.05. Abbreviations: WCB, Weight-control-related Cognition and Behavior; BMI, body mass index.

**Table 4:** Association between baseline centrality and children’s adiposity indicators at the end of the trial.

Degree Centrality	Adiposity Indicators	Model 1 ^a^		Model 2 ^b^	
		Regression Coefficient (95%CI)	*p*	Regression Coefficient (95%CI)	*p*
Out-degree centrality	BMI	−0.047 (−0.076, −0.018)	0.001 *	−0.042 (−0.071, −0.014)	0.004 *
	BMI z score	−0.019 (−0.030, −0.008)	0.001 *	−0.018 (−0.029, −0.007)	0.001 *
In-degree centrality	BMI	−0.052 (−0.084, −0.020)	0.001 *	−0.047 (−0.080, −0.015)	0.004 *
	BMI z score	−0.017 (−0.029, −0.004)	0.008 *	−0.015 (−0.028, −0.003)	0.017 *

^a^ Linear model 1 adjusted for baseline adiposity indicators, group, age, sex, and region. ^b^ Linear mixed model 2 further adjusted for class clustering effect. * *p* < 0.05. Abbreviations: BMI, body mass index.

**Table 5:** Sensitivity analysis of the association between baseline centrality and adiposity indicators at the end of the trial.

Degree Centrality	Adiposity Indicators	Model 1 ^a^		Model 2 ^b^		Model 3 ^c^	
		β (95%CI)	*p*	β (95%CI)	*p*	β/OR (95%CI)	*p*
Out-degree centrality	BMI	−0.036 (−0.066, −0.007)	0.015 *	−0.037 (−0.068, −0.007)	0.018 *	−0.043 (−0.073, −0.014)	0.004 *
	BMI z score	−0.016 (−0.028, −0.005)	0.006 *	−0.018 (−0.030, −0.006)	0.003 *	−0.019 (−0.031, −0.008)	0.001 *
In-degree centrality	BMI	−0.042 (−0.074, −0.010)	0.012 *	−0.045 (−0.079, −0.011)	0.011 *	−0.049 (−0.082, −0.016)	0.004 *
	BMI z score	−0.015 (−0.027, −0.002)	0.026 *	−0.016 (−0.029, −0.002)	0.021 *	−0.016 (−0.029, −0.003)	0.018 *

^a^ Model 1 adjusted for baseline adiposity indicators, group, age, sex, region, class clustering effect, and parental educational level. ^b^ Model 2 adjusted for baseline adiposity indicators, group, age, sex, region, class clustering effect, and parental nutritional status (whether overweight/obese). ^c^ Model 3 adjusted for baseline adiposity indicators, group, age, sex, region, class clustering effect, and whether parents limited children’s screen time. * *p* < 0.05. Abbreviations: BMI, body mass index.

**Table 6:** Sensitivity analysis of the association between baseline centrality and adiposity indicators at the end of the trial.

Degree Centrality	Adiposity Indicators	Model 4 ^a^		Model 5 ^b^		Model 6 ^c^	
		β (95%CI)	*p*	β (95%CI)	*p*	β/OR (95%CI)	*p*
Out-degree centrality	BMI	−0.042 (−0.071, −0.013)	0.005 *	−0.042 (−0.071, −0.014)	0.004 *	−0.042 (−0.071, −0.014)	0.004 *
	BMI z score	−0.018 (−0.030, −0.007)	0.001 *	−0.019 (−0.030, −0.008)	0.001 *	−0.018 (−0.029, −0.007)	0.001 *
In-degree centrality	BMI	−0.047 (−0.080, −0.015)	0.005 *	−0.047 (−0.080, −0.015)	0.004 *	−0.047 (−0.079, −0.015)	0.004 *
	BMI z score	−0.015 (−0.028, −0.003)	0.018 *	−0.015 (−0.027, −0.003)	0.019 *	−0.015 (−0.028, −0.003)	0.017 *

^a^ Model 4 adjusted for baseline adiposity indicators, group, age, sex, region, class clustering effect, and self-efficacy in weight loss. ^b^ Model 5 adjusted for baseline adiposity indicators, group, age, sex, region, class clustering effect, and family support for the intervention program. ^c^ Model 6 adjusted for baseline adiposity indicators, group, age, sex, region, class clustering effect, and school physical-education environment. * *p* < 0.05. Abbreviations: BMI, body mass index.

## Data Availability

Data described in the manuscript, codebook, and analytic code will not be made available because the Peking University Institutional Review Board has not consented to this. In order to access more information on data analysis, contact the corresponding author at whjun@pku.edu.cn.
